# Staff Recognition of Autism and Autistic Traits in Homelessness Services: A Vignette Study

**DOI:** 10.1002/jcop.70095

**Published:** 2026-03-25

**Authors:** Victoria Milner, Georgia Lockwood‐Estrin, Tara Chapple, Alasdair Churchard

**Affiliations:** ^1^ Oxford Institute of Clinical Psychology Training and Research, Isis Education Centre, Warneford Hospital, Headington Oxford UK; ^2^ School of Psychology University of East London London UK; ^3^ Lived Experience Expert Canterbury UK

**Keywords:** access to services, autism, autistic adults, barriers to care, community services, homelessness, reasonable adjustments, staff training

## Abstract

Autistic people are overrepresented among people experiencing homelessness, and better recognition of autism may improve access to homelessness services. This study examined whether staff working in homelessness services identify autism in service users. A total of 203 staff working with people experiencing homelessness in the UK completed an online survey in which they were asked to identify a mental health or neurodevelopmental condition from five vignettes co‐developed with experts by experience. Participants were most accurate at identifying more traditional presentations of autism and least accurate at identifying Emotionally Unstable Personality Disorder (EUPD). Personal or professional connection to, and experience with, autism did not predict accuracy or whether participants said they would make adaptations. These findings suggest that recognition of more nuanced presentations of autism needs to improve. Future research should examine how adaptations are implemented in practice and how service users experience those adaptations.

‘Homelessness’ is defined as the lack of fixed night‐time accommodation (Fazel et al. 2014). The term homelessness covers a range of experiences, from rough sleeping to ‘hidden homelessness’ e.g., sofa surfing (Fazel et al. 2014). The 2023 ‘annual snapshot’ identified 3,898 people rough sleeping across England, and 109,000 households living in temporary accommodation between July and September 2023 (Crisis UK 2024; Department for Levelling Up, H. & C 2024). Figures from January to March 2024 indicate a sharp increase of 19.8% in people and families experiencing homelessness compared to figures from the same period the previous year (Ministry of Housing, C. & L. G 2024), with socio‐political factors such as lack of affordable living and increased cost of living likely playing a role. People experiencing homelessness face increased risk of harm from others, poor mental health and reduced life expectancy (Crisis 2023).

Autism is a neurodevelopmental condition characterised by differences in social communication and interaction, in addition to restricted and repetitive behaviours and/or interests (American Psychiatric Association 2022). Emerging evidence suggests autistic people^1^ are at elevated risk for homelessness. Churchard et al. (2019) interviewed keyworkers of 106 people experiencing homelessness in London with the aim of assessing autistic traits. It was found that 12.3% of the sample had a level of autistic traits consistent with diagnostic criteria. In a similar study conducted in Ireland, 3% of a homeless sample demonstrated a level of autistic traits indicative of meeting diagnostic criteria, with an additional 9% of participants indicating a level of autistic traits that ‘possibly’ indicate autism (Boilson et al. 2023). In contrast, autism prevalence in the general population is between 1% and 2% (Brugha et al. 2011; Zeidan et al. 2022).

There is limited research as to why autistic people are likely overrepresented in homeless populations. The core features of autism may offer some explanation. Differences in social communication and interaction between autistic and non‐autistic peers may lead to increased relationship breakdown and limit social support networks, which in turn may lead to becoming homeless. These social differences may also make it more difficult to live in shared living spaces, adding difficulties getting out of the cycle of homelessness due to the unsuitability of temporary accommodation and limited options for permanent solo living spaces. Preference for sameness may add further challenges when managing change and uncertainty involved in finding a living place. Sensory sensitivities, common for many autistic people, may make available homes unsuitable, therefore limited the options available (Kargas et al. 2019; Osborn and Young 2022). However, it is important to consider these differences through the lens of the social disability model. That is, the increased risk of homelessness for autistic people is likely caused by neurotypical‐biased norms and limited societal empathy and understanding, rather than inherent differences (Woods 2017). For instance, limited understanding of autism and empathy from others may lead to misunderstanding or misinterpretation of behaviours and contribute to the breakdown of suitable housing (Lockwood‐Estrin and Churchard 2024). It is well documented that autistic people face additional barriers to accessing services, likely due to barriers imposed by neurotypical norms and expectations (Mason et al. 2019; Lockwood‐Estrin and Churchard 2024).

In addition to increased risk of becoming homeless, autistic people may face additional barriers when accessing and navigating housing services. For instance, differences in communication and interaction styles and/or challenges understanding implicit social norms likely lead to misunderstanding or misinterpretation of behaviours. In some cases, this may lead to individuals being turned away from services, or having difficulties approaching or engaging with support. Executive functioning differences, such as a need for increased processing time, logical thinking styles, and difficulties managing uncertainty and change have also been noted by autistic people to impair access to services (Stone 2022; Stone et al. 2022). Therefore, enhanced understanding, specialist support, and adaptations are required to improve support for autistic people facing homelessness (Garratt and Flaherty 2023).

Early appropriate support is a key factor for improving outcomes for autistic people (Mozolic‐Staunton et al. 2020; Shaw et al. 2023). A diagnostic label is often the main avenue to appropriate support, yet the evidence suggests that many people experiencing homelessness have above diagnostic threshold levels of autistic traits yet no formal diagnosis. Therefore, recognition of autistic traits by staff working in homelessness services is essential to ensure timely and appropriate support is offered to all.

Autism is often considered a ‘hidden disability’ (Hickey 2023; Shannon 2019). The term ‘hidden disability’ is used to describe autism due to (1) the large heterogeneity in presentations and experiences (Hickey 2023), (2) some autistic traits being ‘internal’ or ‘relational’ and not visible to others, and (3) some autistic people's ability to adapt or mask their autistic traits in social situations (Cook et al. 2021). Emerging evidence has broadened the understanding of the heterogeneity in autism presentations. Evidence has shown that individuals presenting with a nuanced presentation of autism experience missed, mis‐ or delayed diagnoses and support (Kentrou et al. 2024; Young et al. 2018).

People with a better understanding of ‘hidden disabilities’ have a greater ability to recognise them (The Cabinet Office 2020). Autism‐specific training for staff may influence the level of recognition and support offered to autistic people accessing homelessness services. Studies completed in community health teams and youth offending teams have shown that staff members who complete autism‐specific training have greater autism awareness and are more likely to implement adaptations when working with autistic individuals (Ashworth and Tully 2017; Clark et al. 2016). Beyond the work context, existing literature also demonstrates that personal connections with autism (e.g. an autistic family member) significantly increases autism awareness (Dillenburger et al. 2013). It is unclear, however, as to whether training and/or personal connection to autism has this impact in a homelessness context, and/or if it increases the awareness of nuanced presentations of autism.

Autistic people are up to 12 times more likely to experience poor mental health than non‐autistic individuals (Lai et al. 2019). Many autistic people, particularly those with nuanced presentations, have experienced misdiagnosis prior to receiving their autism diagnosis (Fusar‐Poli et al. 2022; Hull et al. 2020a; Kentrou et al. 2024). For instance, a recent large‐scale study found 0.7% of autistic adults received a diagnosis of emotionally unstable personality disorder (EUPD) prior to their autism diagnosis; however, the EUPD diagnosis only remained stable for 52% of these individuals following autism diagnosis. Similarly, 1% had a preceding diagnosis of a psychotic disorder; however, this diagnosis only remained stable for 45.4% of participants (total *n* = 72,331) (Martini et al. 2025). EUPD is characterised by a pattern of intense and unstable relationships, a disturbed sense of self, impulsive behaviours, difficulties with emotion regulation and chronic feelings of emptiness (World Health Organisation 2022). Psychotic disorders are characterised by an altered sense of reality, such as hallucinations or delusions, in addition to a range of behaviours including disorganised thinking and behaviours, blunted affect or psychomotor disturbances (World Health Organisation 2022). Misdiagnosis for autistic people may be explained by a misinterpretation of autistic features such as difference in emotional expression and non‐verbal behaviours, in addition to differences in social interaction preferences. Staff working in homelessness service may be more familiar with mental health presentations due to elevated prevalence within populations experiencing homelessness, with estimated prevalences of 23.1% for personality disorders and 12.7% for psychosis (Fazel et al. 2008). Therefore, recognition of autism and related needs in homelessness services may be overshadowed by cooccurring mental health diagnoses.

Taken together, the heterogenous presentations of autism, autistic people's vulnerability to poor mental health and the overrepresentation of mental health difficulties in homelessness services, in combination with limited access to specific autism training, may contribute to poor recognition of and support for autism‐related needs.

## 1 Aims and Hypotheses

This study aims to explore the extent to which staff working in homelessness services identify autism in service users, and whether staff consider making adaptations to service users support.

The hypotheses are as follows:

1. Staff working in homelessness services will identify autism in service user vignettes less accurately than other diagnoses (e.g., psychosis, personality disorder).
2. The accurate identification of autism will be predicted by prior experience and/or autism‐specific training.
3. Prior experience and/or autism‐specific training predicts the presence of adaptations to support for autistic service users.

## 2 Materials and Method

### 2.1 Participants

Staff with more than 6 months experience working in homelessness services (third sector, government agencies, or housing associations) in the UK, with face‐to‐face contact with service users were invited to participate via the researchers’ networks, social media, and direct contact with services via email.

A power analysis was conducted using GPower version 3.1.9.2. A medium Cohen's *f* effect size of 0.25 was used based on the results of Tsirgiotis et al. (2022) who reported Cohen's *f* of 0.24–0.62 (converted from Log Odds Ratios). The lower end of this range was chosen to allow for medium effect sizes to be detected in this study. To achieve power of 0.80 for an effect size of 0.25, a sample size of 200 participants was required.

A total of 314 potential participants expressed interest in completing the study between January and October 2024. Of these, 261 participants commenced the main survey; however, 58 were removed due to incompletion. Therefore, a final sample of 203 participants were included in the analysis. Demographic data can be found in Table 1.

**Table 1:** Participant demographics.

Variable		*N* (%)
**Gender**	Male (including trans man)	59 (29%)
	Female (including trans woman)	139 (68.5%)
	Non‐binary/gender fluid	2 (1%)
	Prefer not to say	2 (1%)
	Missing	1 (0.5%)
**Mean age**		41.5 years (SD = 11.9)
**Age range**		20–69 years
**Ethnicity** a	Asian	2 (1%)
	Black African	3 (1.5%)
	Mixed/Multiple Ethnicities	6 (3%)
	White British	169 (83.3%)
	White Other	22 (10.7%)
	Prefer not to say	1 (0.5%)
**UK nation**	England^b^	185 (91%)
	Wales	4 (2%)
	Scotland	1 (0.5%)
	Northern Ireland	1 (0.5%)
	Not Specified	12 (6%)
**Service type**	Third Sector/Charitable	142 (73%)
	Government/Local Authority	51 (25%)
	NHS	6 (3%)
	Housing Associations	3 (1.5%)
	Other not specified	1 (0.5%)
**Mean time working in homelessness service**		7.12 years (SD = 7.23) Range = 0.5–30 years
**Autism diagnosis**	Formal Diagnosis	3 (1.5%)
	Self‐Identify	9 (4.4%)
	Not autistic	181 (89.2%)
	Prefer not to say	9 (4.4%)
	Missing	1 (0.5%)
**Other neurodivergent (ND) Diagnosis**	No other ND diagnosis	156 (76.8%)
	Formal ND diagnosis	29 (14.3%)
	Self ND diagnosis	28 (13.8%)
	Prefer not to say	6 (3%)
**Known autistic person in personal life**	Close family member(s)	72 (35%)
	Extended family member(s)	37 (18.2%)
	Friend(s)	83 (40.9%)
	Romantic partner(s)	19 (9.4%)
	Colleague(s)	47 (23.2%)
	Other	12 (5.9%)
	Prefer not to say	5 (2.5%)
	I do not know any autistic people in my personal life	41 (20.2%)
**Experience working with autistic service users**	Yes	175 (86.2%)
	No	16 (7.9%)
	Unsure	12 (5.9%)
**Average number of autistic service users worked with** c		28.92 (SD = 80.94)
		Range = 0–1000 service users
**Autism specific training**	Yes	102 (50.2%)
	No	101 (49.8%)

^a^ Other ethnicities were shown to participants; however, only the ethnicities selected are presented here.

^b^ Representation was present from all English regions; full breakdown can be found in Appendix 6.

^c^ Participants were asked to estimate the number of service users they have worked with, but it did not specify in their current role, therefore some participants have estimated across their careers.

### 2.2 Design

Using vignettes allows for the amalgamation of service user characteristics, whilst maintaining anonymity. This design has been utilised in studies examining the recognition of autism (e.g. Whitlock et al. 2020). A randomised design was used to present five vignettes to participants and measure the effect of vignette type on the likelihood of diagnosis.

## 3 Materials and Measures

### 3.1 Vignette Development

An initial vignette framework was developed between authors (VM and GLE) to ensure consistency across the vignettes. The framework included a statement for the start of each vignette (‘You are working with [name] who is in their 30s and is currently experiencing homelessness. You have received a referral from the local authority as their temporary accommodation arrangement has broken down and they are at risk of rough sleeping.’), and five descriptors to include for each:

1. The problem(s) with temporary accommodation
2. The key event first leading to homelessness.
3. The first contact with homelessness services from the perspective of the service user.
4. The first contact with the homelessness service from the perspective of ‘your’ (the reader's) colleague.
5. The perspective of the current contact from the person reading the vignette.

This framework was finalised with an autistic woman who works in a homelessness service and who has lived experience of homelessness, and the final author (AC).

Vignettes were subsequently co‐developed with (1) staff in homelessness services with experience of working with autistic service users, (2) researchers working in the field of homelessness and autism, and (3) service users with a personality disorder, psychosis, or an autism diagnosis, in addition to experience of homelessness, to enhance face validity (Evans et al. 2015; Gould 1996). Two autism vignettes were included to capture the ‘nuanced’ presentation of autism based on the ‘female phenotype’ (Hull et al. 2020a, 2020b), in addition to more traditional presentations of autism. Psychosis and Emotionally Unstable Personality Disorder (EUPD) presentations were chosen in discussion with staff working in homelessness services, and due to evidence of misdiagnosis of psychosis/EUPD for autistic individuals (Belcher et al. 2023; Lai and Baron‐Cohen 2015; Van Schalkwyk et al. 2015). All experts by experience were paid £15.

To enhance content validity an iterative process was followed to ensure majority agreement on the presentation described. An online survey was created via Qualtrics including the five vignettes presented in a random order, and an open‐text box after each in which participants were asked to ‘Please state the diagnosis (if any) you think this service user may have. Please only enter one diagnostic label, if you do not know or are not sure please state ‘not sure’. If you think the service user has no psychological diagnosis, then please put ‘none’.

The validation survey was circulated to clinical psychologists (including DClinPsych trainees) through the researchers’ networks and social media. Data were preliminarily checked after 16 responses to the validation survey revealing only 1/16 participants accurately labelled the EUPD vignette (the other vignettes ranged from 70% to 100% accuracy; see Table 2). The EUPD vignette was subsequently amended in consultation with a clinical psychologist working in a personality disorder service and a trainee clinical psychologist with experience working with this population. Following these consultations, traits of impulsivity, emotional lability, relationship breakdown and risky behaviour were emphasised within the vignette and the updated EUPD vignette was added to the validation survey.

**Table 2:** Vignette development survey participant responses.

Vignette	Label	*N* (%)
Traditional autism	**Autism** a	48 (85.7%)
	No diagnostic label	8 (14.3%)
Nuanced autism	**Autism** a	39 (69.6%)^b^
	None/not sure	13 (23.2%)
	‘interpersonal difficulties linked to trauma’	1 (1.8%)
	‘anti‐social personality disorder’	1 (1.8%)
	Acquired brain injury	1 (1.8%)
Psychosis	**Psychosis**	32 (76.8%)
	None/not sure	9 (16.1%)
	Complex Trauma	1 (1.8%)
	Autism	3 (5.4%)
EUPD	**EUPD**	26 (65%)^c^
	‘insecure attachment’	1 (2.5%)
	‘anxiety/social anxiety’	2 (5%)
	Autism	2 (5%)
	none	4 (10%)
	‘trauma’/‘CPTSD’/‘Childhood abuse’	5 (12.5%)
No additional diagnosis	**None**	45 (80.4%),
	Low mood/‘circumstantial’ depression	11 (19.6%)

*Note:* The intended/accurate answer is in bold for each vignette. All percentages have been rounded to the nearest one decimal point.

Abbreviation: EUPD, emotionally unstable personality disorder.

^a^ Iterations of autism were suggested, including ‘ASD’ and ‘autism spectrum condition’.

^b^ The authors deemed this acceptable as the concept of nuanced autistic traits (based on the notion of a female autism phenotype; Hull et al. 2020) has emerged in recent years and likely explains lack of recognition.

^c^ Due to debate regarding the overlap between trauma and EUPD (Lewis and Grenyer 2009), and research evidencing the misdiagnosis between EUPD and autism (Fusar‐Poli et al. 2022), 65% was deemed an acceptable threshold.

Forty psychologists completed the second phase of the validation survey. The updated survey was not sent to psychologists who had previously been invited, therefore, it is probable that these responses were new participants; however, this cannot be guaranteed as participation was anonymous. As the other vignettes remained the same, data from both validation phases (*n* = 56) have been included for all but the EUPD vignette (*n* = 40).

### 3.2 Online Survey

For the main survey, following the information sheet and consent form (see Appendix 4), five vignettes were presented to participants in a randomised order via Qualtrics to reduce order effects. An open text box with the following instructions was presented after each vignette ‘please state the diagnosis (if any) you think this service user may have. Please only enter one diagnostic label, if you do not know or are not sure please state ‘not sure’. If you think the service user has no psychological diagnosis, then please put ‘none’’. Participants were then asked to rate the likelihood that the service user would meet diagnostic threshold for a list of mental health and neurodevelopmental conditions on a visual analogue scale (VAS) ranging from ‘0’ – not at all likely, to ‘100’ – extremely likely. The list of diagnoses was decided in discussion with experts by experience and included: autism, depression, generalised anxiety disorder, personality disorder, psychosis, substance misuse. These options were selected to include choices beyond those represented in the vignettes, to minimise participants learning or assuming the ‘correct’ response. Options were presented in alphabetical order.

For each vignette, participants were asked if they would adapt the provision they provide using yes/no/unsure options. If the participant indicated ‘yes’ or ‘unsure’ the participant was asked ‘what adaptations to support would you consider for this service user’ in an open text box.

**Demographics:** Participants were asked to state their age in years, gender and ethnicity. Participants were then asked to state the type of service they worked in from multiple choice options and their job title, number of years of experience in homelessness services, UK county in open text box.

**Autism experience:** Participants were asked whether they have a formal or self‐identify of autism and/or other neurodivergent diagnosis. Participants were asked if they know anyone who has an autism diagnosis in their personal life, and whether they have worked with autistic service users and to estimate how many to the nearest 10. They were asked whether they have received specific autism training and to provide details about the duration, context, content and timing of the training.

**Psychosis and EUPD experience:** the same questions outlined in the section above were adapted to ask participants about psychosis and EUPD.

A full copy of the survey can be found in Appendix 4.

### 3.3 Procedure

Ethical approval was received from University of Oxford Medical Sciences Interdivisional Research Ethics Committee (R90351/RE001). Participants were invited to take part via email, social media, and posters (see Appendix 2). To reduce the likelihood of artificial or ‘bot’ data, participants were initially directed to an ‘expression of interest’ survey in which they were asked to include their job role, their place of work and a workplace email address (See Appendix 3). If the researchers were unsure about the authenticity of the contact details, the potential participant was contacted to confirm they met inclusion criteria. A link for the online survey was then sent directly to the participant via email.

Once participants accessed the main survey link, they were shown an information sheet and consent form, followed by the vignette block, the demographic block, the autism experience block and the psychosis/EUPD block in order. Once data collection was closed (October 2024), all participants were sent a debrief email (see Appendix 5).

#### 3.3.1 Ethical Considerations

Participants were not informed that autism is the focus on the study at the start of the study. This was essential as if participants were aware that the aim of the study was to assess their ability to identify autism, they would likely be vigilant to perceived traits and answers may be biased. Participants were told the study aimed to investigate staff members’ ability to identify service user needs. Participants were debriefed via email (See Appendix 5). Participants were given a £10 voucher upon completion of the survey.

### 3.4 Data Analysis

Analysis was conducted using SPSS Version 29.0.2.0.

Data were manually checked for fraudulent responses. If participants did not provide open text responses during the vignette block of questions, their answers in the demographic and experience blocks were examined to determine whether responses appeared realistic and appropriate. If participants did not accurately respond to the ‘attention check’ question, their open text responses were examined to determine engagement with the survey. All data were deemed to be appropriate and realistic.

#### 3.4.1 Continuous Accuracy Scores

Following the original proposed data analysis plan, discrepancy variables were created from the VAS for each of the ‘correct’ answers for each vignette by subtracting the participant's score from 100. As the VAS were not forced responses, if a participant had left it blank but had demonstrated their understanding of the VAS in other vignettes, their discrepancy score was assumed to be 100 (indicating a VAS of 0). These scores were imputed manually for each participant. Lower discrepancy scores indicate greater accuracy.

#### 3.4.2 Dichotomous Accuracy Scores

After initial data analysis was complete, feedback from colleagues and peer reviewers highlighted that continuous data (described below) may be describing participant *confidence* in diagnostic labels rather than accuracy. Furthermore, it was noted that as participants were selecting labels from a ‘forced choice’ list, this likely influenced their answers. Therefore, the authors manually coded accuracy as accurate/not accurate variables based on participant open‐text responses.

For vignette one and two, any open‐text response including a label describing autism (i.e. autism, ASD, Asperger's, autistic) was coded as ‘1’ or ‘accurate’. All other open‐text labels were coded as ‘0’ or ‘not accurate’. Open‐text responses were coded as accurate for vignette three if they included any mention of psychosis, schizophrenia or paranoia. For vignette four, open‐text responses mentioning any personality disorder (including abbreviations such as ‘PD’) were coded as accurate.

#### 3.4.3 Hypothesis One

In line with the original data analysis plan, to compare variation in accuracy (continuous DV: discrepancy score) across vignettes (categorical IV: autism, psychosis, PD), a one‐way repeated measures ANOVA was conducted. Pairwise comparisons were analysed, with Bonferroni corrections for multiple comparisons.

To address the likelihood that discrepancy scores likely measure *confidence* rather than accuracy, additional analysis was conducted utilising the dichotomous accuracy scores. To test overall difference in accuracy across vignettes, a Cochran's Q test was conducted. Pairwise McNemar's tests were subsequently conducted to compare each vignette directly. Binominal McNemar's test are reported due to counts less than 25 in some cells. Bonferroni corrections were applied to pairwise comparisons.

#### 3.4.4 Hypothesis Two

Multiple linear regressions were conducted to determine whether prior experience/training (IVs: number of years working in homelessness services (continuous variable), participant's autism diagnosis (dichotomous autistic/not autistic variable), whether they have a known autistic person in their own life (dichotomous yes/no variable), the number of autistic people worked with (continuous variable), and autism‐specific training received (dichotomous yes/no variable)) predict accuracy on the autism vignettes (continuous DV: discrepancy score).

#### 3.4.5 Hypothesis Three

Binominal logistic regressions were completed to determine whether prior experience/training (IVs: number of years working in homelessness services, participant's autism diagnosis, personal experience (e.g. autistic family, friend, colleague), professional experience, the number of autistic people worked with, and autism‐specific training received) predict whether the participant will adapt support (categorical DV: yes/no) for autistic service users depicted in the vignettes.

## 4 Results

### 4.1 Hypothesis One: Staff Working in Homelessness Services Will Identify Autism in Vignettes Less Accurately Than Other Diagnoses (e.g., Psychosis, Personality Disorder)

All assumptions were met with the exception of the assumption of sphericity, Huynh‐Feldt correction was applied (ε = 0.97).

Discrepancy scores were statistically significantly different across vignettes, *F*(2.91, 587.61) = 36.24, *p* < 0.001, partial η^2^ = 0.152. Post‐hoc analysis with Bonferroni corrections revealed that participants were significantly more accurate at labelling the diagnosis presented in vignette one (‘traditional autism’) compared to vignette two (‘nuanced autism’, mean difference = −9.41, 95% CI [−15.53 to −3.30], *p* < 0.001), vignette three (psychosis, mean difference = −10.16, 95% CI [−16.41 to −3.90], *p* < 0.001) and vignette four (EUPD, mean difference = −26.35, 95% CI [−33.73 to 18.97], *p* < 0.001).

Participants were significantly more accurate at labelling vignette two (‘nuanced autism’) compared to vignette four (EUPD, mean difference = −16.94, 95% CI [−24.29 to −9.59], *p* < 0.001), but not vignette three (psychosis, mean difference = −0.74, 95% CI [−7.41 to 5.92], *p* = 1.00). Participants were significantly more accurate at labelling vignette three (psychosis) compared to vignette four (EUPD, mean difference = −16.19, 95% CI [−23.39 to −8.99], *p* < 0.001). Labels and accuracy for each vignette can be found in Table 3.

**Table 3:** Percent accurate and other diagnostic label(s) provided by participants separated by vignette number.

		*N* (%)
**Vignette 1**	Addiction	1 (0.5%)
	ADHD	3 (1.5%)
	**Autism** d	161 (79.3%)
	Anxiety^b^	18 (8.9%)
	Depression	3 (1.5%)
	Developmental disorder	2 (1%)
	Dyslexia	1 (0.5%)
	Neurodivergent^d^	6 (3%)
	None	1 (0.5%)
	Not Sure	16 (7.9%)
	Personality disorder^e^	1 (0.5%)
	Social and emotional challenges	1 (0.5%)
	Trauma^c^	2 (1%)
	**Mean discrepancy score**	**= 25.50 (SD** = **26.16)**
**Vignette 2**	Addiction	1 (0.5%)
	ADHD	8 (3.9%)
	Anxiety^b^	13 (6.4%)
	**Autism** a	127 (62.6%)
	Bipolar	2 (1%)
	Depression	2 (1%)
	Developmental disorder	1 (0.5%)
	Dyslexia	1 (0.5%)
	Learning difficulty	1 (0.5%)
	Learning disability	1 (0.5%)
	Neurodivergent^d^	5 (2.5%)
	None	3 (1.5%)
	Not Sure	30 (14.8%)
	Personality disorder^e^	16 (7.9%)
	Psychosis^f^	4 (2%)
	Trauma^c^	2 (1%)
	**Mean discrepancy score**	**= 34.91 (SD** = **30.41)**
**Vignette 3**	ADHD	4 (2.0%)
	Anxiety^b^	3 (1.5%)
	Autism^a^	7 (3.4%)
	Bipolar	2 (1%)
	Depression	3 (1.5%)
	Mental Health	4 (2%)
	None	1 (0.5%)
	Not Sure	29 (14.3%)
	Personality disorder^e^	21 (10.3%)
	**Psychosis** f	134 (66%)
	Substance misuse	3 (1.5%)
	**Mean discrepancy score**	**= 35.66 (SD** = **30.09)**
**Vignette 4**	Adjustment disorder	1 (0.5%)
	Anxiety^b^	28 (13.8%)
	Attachment Disorder^g^	13 (6.4%)
	Autism^a^	25 (12.3%)
	Bipolar	2 (1%)
	Depression	17 (8.4%)
	None	4 (2%)
	Not Sure	29 (14.3%)
	**Personality Disorder** e	79 (38.9%)
	Psychosis^f^	5 (2.5%)
	Self‐Harm	1 (0.5%)
	Suicidality	1 (0.5%)
	Trauma^c^	29 (14.3%)
	**Mean discrepancy score**	**= 51.85 (SD** = **35.80)**
**Vignette 5**	Anxiety^b^	43 (21.2%)
	Depression	79 (38.9%)
	**None**	82 (40.4%)
	Not Sure	12 (5.9%)
	Trauma^c^	1 (0.5%)

*Note:* some participants provided more than one label for each vignette therefore the *n* > 203 and the percentages will be > 100%.

Correct response is in bold for each vignette.

Abbreviations: ADHD, attention deficit hyperactivity disorder; SD, standard deviation.

^a^ Responses included all descriptions of autism: ‘autism spectrum condition’, ‘autism spectrum disorder’, ‘ASD’, ‘Aspergers’ ‘possible autism’ and ‘autistic’.

^b^ Responses included ‘anxiety disorder’, ‘anxiety’, ‘generalised anxiety disorder’, ‘GAD’, ‘possible anxiety’, ‘Social Anxiety’ and ‘severe anxiety’.

^c^ Responses included ‘Complex PTSD’ and ‘trauma’.

^d^ Responses included ‘neurodiverse’ and ‘neurodivergent’.

^e^ Responses included ‘Narcissistic Personality Disorder’, ‘Borderline Personality Disorder’, ‘EUPD’ and ‘Personality Disorder’.

^f^ Responses included ‘psychosis’, ‘schizophrenia’, ‘paranoia’, ‘paranoid psychosis’, ‘drug induced psychosis’, ‘schizoaffective disorder’, ‘psychotic disorder’ ‘first episode psychosis’.

^g^ Responses include ‘anxious attachment’, ‘attachment difficulties’, ‘attachment disorder’, ‘attachment issues’, ‘disordered attachment style’.

A similar pattern of findings was found using the dichotomous accuracy scores. Cochran's Q test indicated significant differences across the five conditions, Q(4, *N* = 203) = 117.63, *p* < 0.001. Post‐hoc McNemar's tests with Bonferroni correction (α = 0.005) showed that participants endorsed significantly more ‘accurate’ responses in the traditional autism vignette compared to the nuanced autism vignette (exact binomial test, *p* < 0.001), the psychosis vignette (exact binomial test, *p* < 0.001), the EUPD vignette (exact binomial test, *p* < 0.001).

Participants were significantly more accurate for the nuanced autism compared to the EUPD vignette (exact binomial test *p*< 0.001) but not the psychosis vignette (exact binomial test, *p* = 0.550), and more accurate for the psychosis compared to EUPD vignette (exact binomial test *p*< 0.001).

### 4.2 Hypothesis Two: The Accuracy of Identification of Autism Will Be Predicted By Prior Experience, and/or Autism‐Specific Training

**Vignette one**

Assumption checks for the linear regression analysis found that there was independence of residuals, as assessed by a Durbin–Watson statistic of 1.946. P–P plots were visually inspected, and data were deemed normally distributed. One data point had a high leverage point (0.77); however, it was not an outlier, nor a high influential point, therefore, it was included in the analysis.

The model was not statistically significant, *F*(6, 173) = 1.084, *p* = 0.374, adj *R* ^2^ = 0.3%; prior experience (number of years working in homelessness services, participant's autism diagnosis, known autistic person in persona life, number of autistic people worked with, and autism‐specific training received) did not predict accuracy (discrepancy scores) for vignette one.

**Vignette two**

Assumption checks for the linear regression analysis found that there was independence of residuals, as assessed by a Durbin‐Watson statistic of 2.109. P–P plots were visually inspected, and data were normally distributed. One data point had a high leverage point (0.77) and high influential point ( > 1); therefore, it was excluded in the analysis.

The model was not statistically significant, *F*(6, 172) = 0.551, *p* = 0.769, adj *R* ^2^ = −1.5%; prior experience (number of years working in homelessness services, participant's autism diagnosis, known autistic person in persona life, number of autistic people worked with, and autism‐specific training received) did not predict accuracy (discrepancy scores) for vignette two.

Standardised beta coefficients for both vignettes can be found in Table 4.

**Table 4:** Linear regression analysis to address hypothesis two.

		Vignette 1	Vignette 2
Overall model		*F*(6, 173) = 1.084, *p* = 0.374, adj *R* ^2^ = 0.3%	*F*(6, 172) = 0.551, *p* = 0.769, adj *R* ^2^ = −1.5%
Professional experience			
	Autism‐specific training (yes/no)	−0.067	−0.016
	Worked with autistic people (yes/no)	−0.048	.043
	Number of autistic people worked with	−0.047	−0.038
	Years worked in homelessness services	.010	−0.014
Personal experience			
	Autistic/not autistic	−0.126	−0.105
	Known autistic person in their life (yes/no)	−0.061	−0.060

*Note:* None of the included coefficients were significant predictors of accuracy.

### 4.3 Hypothesis Three: Prior Experience and/or Autism‐Specific Training Predicts the Presence of Adaptations to Support for Autistic Service Users

Summary statistics can be found in Table 5.

**Table 5:** Participant response to ‘Would you make adaptations to the support provision you provide this service user’ separated by vignette.

		*N* (%)
**Vignette 1 – autism (traditional)**	Yes	170 (83.7%)
	No	12 (5.9%)
	Unsure	21 (10.3%)
**Vignette 2 – autism (nuanced)**	Yes	169 (83.3%)
	No	9 (4.4%)
	Unsure	19 (9.4%)
	Missing	6 (3%)
**Vignette 3 – psychosis**	Yes	163 (80.3%)
	No	10 (4.9%)
	Unsure	30 (14.8%)
**Vignette 4 – EUPD**	Yes	147 (72.4%)
	No	14 (6.9%)
	Unsure	36 (17.7%)
	Missing	6 (3%)
**Vignette 4 – control**	Yes	75 (36.9%)
	No	86 (42.4%)
	Unsure	39 (19.2%)
	Missing	3 (1.5%)

**Vignette one**

Assumption checks for the logistic regression found there were nine standardised residuals with a value of −2.615 to –5.281 standard deviations; these cases were checked and were included in the analysis. All other assumptions were met.

The logistic regression model was not statistically significant, χ^2^(8) = 9.446, *p* = 0.306. The model explained 11.1% (Nagelkerke *R* ^*2*^) of the variance in adaptations made and correctly classified 86.1% of cases. Sensitivity was 98.7%, specificity was 4.3%, positive predictive value was 87.06% and negative predictive value was 33.33%.

Participant's own autism diagnosis, known autistic person in persona life, number of autistic people worked with, and autism‐specific training received did not predict whether staff would make adaptations to support for the service user depicted in vignette one. However, the number of years working in homelessness services (odds ratio = 0.60) and whether a person has worked with an autistic person or not (odds ratio = 12.57) were significant independent predictors of whether adaptations were made.

**Vignette two**

Assumption checks for the logistic regression found there were nine standardised residuals with a value of −2.56 to −4.64 standard deviations; these cases were check and were included in the analysis. All other assumptions were met.

The logistic regression model was not statistically significant, χ^2^(8) = 5.86, *p* = 0.663. The model explained 4.7% (Nagelkerke *R* ^*2*^) of the variance in adaptations made and correctly classified 100% of cases. Sensitivity was 100%, specificity was 0%, positive predictive value was 85.55% and negative predictive value was 0%.

Prior experience (number of years working in homelessness services, participant's autism diagnosis, known autistic person in persona life, number of autistic people worked with, and autism‐specific training received) did not predict whether staff would make adaptations to support for the service user depicted in vignette two.

Table 6 summarises the odds ratio and confidence intervals for each vignette.

**Table 6:** Logistic regression analysis to address hypothesis two.

		Odds ratios (CI)	
		Vignette 1	Vignette 2
Overall model		χ^2^(8) = 9.446, *p* = 0.306. Nagelkerke *R* ^*2*^ = 11.1%	χ^2^(8) = 5.86, *p* = 0.663. Nagelkerke *R* ^*2*^ = 4.7%
Professional experience			
	Autism‐specific training (yes/no)	1.36 (0.51–3.63)	.705 (0.27–1.83)
	Worked with autistic people (yes/no)	12.57 (1.885–83.76)*	2.64 (0.41–17.19)
	Number of autistic people worked with	1.07 (0.71–1.63)	1.38 (0.90–2.11)
	Years worked in homelessness services	0.60 (0.36–0.98)*	1.06 (0.70–1.61)
Personal experience			
	Autistic/not autistic	0.70 (0.14–3.57)	1.60 (0.19–13.14)
	Known autistic person in their life (yes/no)	0.51 (0.11–2.40)	0.864 (0.24–3.09)

* For vignette one, professional experience of working with autistic people and the years worked in homelessness services were significant independent predictors.

## 5 Discussion

There is evidence of a higher prevalence of autistic traits in populations experiencing homelessness, compared to the general population. Autistic people experiencing homelessness may face additional barriers when accessing and navigating services, requiring additional support. This study aimed to explore whether staff working in homelessness services were able to accurately identify autism in vignettes of service users experiencing homelessness.

The results showed that compared to vignettes depicting service users with nuanced presentations of autism, psychosis and emotionally unstable personality disorder (EUPD), participants were more accurate at identifying traditional presentations of autism, refuting our initial hypothesis. Participants had similar levels of accuracy when identifying nuanced presentations of autism and psychosis in the vignettes. Participants were least accurate at identifying EUPD in the vignettes. Regression analyses revealed that participants’ accuracy at identifying ‘traditional’ and ‘nuanced’ presentations of autism in the vignettes, and whether they would make adaptations when working with these service users, were not predicted by personal or professional experience or training; thus, not supporting our second and third hypotheses. The majority of participants stated that they would make adaptations for all of the service users depicted in all but the ‘control’ vignette, suggesting that staff working in homelessness services are adept at recognising service users’ needs and considering needs‐led support regardless of diagnostic status.

Interestingly, participants were significantly more accurate at identifying ‘traditional’ compared to ‘nuanced’ autism. This reflects findings from existing literature highlighting nuanced presentations are less likely to be captured using current diagnostic tools and are likely missed due to sociocultural and gendered clinician biases (Lai et al. 2023; Tsirgiotis et al. 2024). While the authors did not examine the role of service user gender in this study, it is important to consider the confounding impact of gender and nuanced presentations. Autistic women are more likely to experience hidden homelessness (Lockwood Estrin et al. 2022), meaning the current estimated prevalence of homelessness are likely under representative. A 2022 collaboration of 26 stakeholders identified increased staff awareness as a key priority for improving service provision and preventing cycles of homelessness for autistic women (Lockwood Estrin et al. 2022). The findings of the current study indicate the opportunity to increase staff awareness of nuanced presentations of autism.

Personal and professional connection and experience were not found to predict autism awareness in the current study. Existing literature have found a significant link between knowing an autistic person and awareness of the Autism Act, a legal framework protecting the rights of autistic individuals (Autism Act 2009; Dillenburger et al. 2013). The insignificant finding of the current study may be explained by a growing awareness of autism in the general population. Dillenburger et al. (2013) explored autism awareness in the general population in Northern Ireland via online survey and found 80% of participants indicated high levels of autism awareness. Since the implementation of the Autism Act in 2009, there has been an increasing coverage of autism in popular culture, education, social care and healthcare contexts (Fontes and Pino‐Juste 2022; Kirby and Payne 2023). Therefore, it is possible that the accuracy of diagnostic labelling in this study reflects a general increase in awareness, rather than awareness stemming from personal or professional knowledge and experience.

In addition to increased autism awareness in the general population, The Autism and Homelessness Toolkit first developed by Churchard et al. (2019) provides an introduction to autism for staff working in homelessness services. The second edition (Lockwood‐Estrin and Churchard 2024) introduces the topic of autism and gender, and outlines traits more likely to be observed in autistic women based on the current literature. It is possible that the roll out of this toolkit improved participants awareness and recognition of autism in this study explaining the significantly more accurate recognition of traditional autism; however, this was not formally assessed.

Personal and professional knowledge and experience also did not predict whether a person would make adaptations in the overall regression model. However, for vignette one, the binary variable of whether someone had worked with an autistic person or not was a significant independent predictor with large odds ratio; those who had worked with an autistic person were significantly more likely to make adaptations for the individuals with a traditional presentation of autism depicted in vignette one. This finding is in contrast with the non‐significant findings for vignette two and may reflect enhanced confidence for making adaptations for traditional presentations which is not aligned with non‐traditional presentations. Studies exploring the impact of training for administrative staff in a healthcare setting found an increase in adaptations being made for autistic service users following training (Clark et al. 2016). Although the majority of participants in the current study indicated they would make adaptations for the hypothetical service users, it would be useful to explore whether this reflects service users’ experience in reality, and whether differences occur when supporting different presentations of autism. The current study does not examine whether completing training or having a personal connection predicts a person's confidence or likelihood of implementing the proposed adaptations; a binary yes/no answer may be reductive and not capture the nuance of this topic. It is possible that participants’ responses to whether or not they would make adaptations reflect an ‘in an ideal world’ scenario, and in reality, there may barriers to implementing change.

Although not the focus of this study, it is important to note that the significantly lower accuracy in labelling EUPD may reflect a movement away from personality disorder terminology towards a trauma‐informed understanding of difficulties (Lewis and Grenyer 2009). The alternative labels provided for this vignette listed in Table 3 support this; approximately 21% of participants suggested trauma or attachment difficulties. A further 14.3% suggested autism as a label for the EUPD vignette, possibly reflecting the misdiagnosis of autism as a personality disorder, which has been the reality for many autistic people (Fusar‐Poli et al. 2022).

## 6 Strengths and Limitations

The authors co‐developed vignettes with people with lived experience of homelessness and one or more of the depicted diagnoses. The authors also endeavoured to control for gender in the vignettes by using gender neutral pronouns and names. As with all vignette studies, the ecological validity is limited. Additionally, a response bias might be present; indication of whether participants would make adaptations for the service user be inflated due to desirability effects. A large proportion of the current sample had personal experience of autism, almost 30 times more than current estimated prevalence of autism (Zeidan et al. 2022), in addition to a large range of professional experience. It is possible that the level of knowledge in this sample is not representative of the general population; results should be interpreted with that in mind. Finally, there was no objective measure of autism knowledge included in the study. It is possible that despite completing autism training, participant's knowledge did not improve.

## 7 Future Research and Recommendations

Further research is needed to explore the implementation of adaptations in reality. This research may explore whether autistic service users require more, or more specific, adaptations than non‐autistic service users. It would also be useful to investigate the impact of adaptations from both a staff and service user perspective, examining any discrepancies. Although we did not find in this study that training predicted recognition or accommodations, it would be useful to explore changes to staff confidence, awareness and behaviour pre and post training.

## 8 Conclusions

Participants were most accurate at identifying traditional presentations of autism, compared to nuanced autism, psychosis and EUPD. Personal and professional connection and experience did not predict accuracy nor whether participants would make adaptations. This can possibly be explained by growing autism awareness in the general population. Improvements to the recognition of nuanced autism is needed. Future research could explore the implementation of adaptations in reality, and service users’ experience of adaptations.

## Author Contributions

Victoria Milner, Georgia Lockwood‐Estrin, Tara Chapple and Alasdair Churchard conceived and designed the project and developed the research materials. Victoria Milner acquired, analysed and interpreted the data and wrote the initial draft. Victoria Milner, Georgia Lockwood‐Estrin, Tara Chapple, Alasdair Churchard reviewed and edited the manuscript. Georgia Lockwood‐Estrin and Alasdair Churchard supervised Victoria Milner. Alasdair Churchard acquired funding for the project.

## Ethics Statement

Ethical Approval was obtained from University of Oxford Central University Research Ethics Committee (CUREC), ethics number: R90351/RE001.

## Data Availability

The data that support the findings of this study are available from the corresponding author upon reasonable request. The data that support the findings of this study are available on request from the corresponding author. The data are not publicly available due to privacy or ethical restrictions.

## Endnotes

1 Identity‐first language (autistic person) is used throughout this paper to reflect the preference of the autistic community (Kenny et al. 2016).
